# The Ratio of Factor VIIa:Tissue Factor Content within Microvesicles Determines the Differential Influence on Endothelial Cells

**DOI:** 10.1055/s-0039-1688934

**Published:** 2019-05-15

**Authors:** Yahya Madkhali, Sophie Featherby, Mary E. Collier, Anthony Maraveyas, John Greenman, Camille Ettelaie

**Affiliations:** 1 Department of Biomedical Sciences, University of Hull, Hull, United Kingdom; 2 Department of Medical Laboratories, College of Applied Medical Sciences, Majmaah University, KSA, Al Majmaah, Saudi Arabia; 3 Department of Cardiovascular Sciences, University of Leicester, Glenfield General Hospital, Leicester, United Kingdom; 4 Division of Cancer–Hull York Medical School, University of Hull, Hull, United Kingdom

**Keywords:** tissue factor, microvesicles, protease-activated receptor-2, apoptosis, cell proliferation, factor VIIa

## Abstract

Tissue factor (TF)-positive microvesicles from various sources can promote cellular proliferation or alternatively induce apoptosis, but the determining factors are unknown. In this study the hypothesis that the ratio of fVIIa:TF within microvesicles determines this outcome was examined. Microvesicles were isolated from HepG2, BxPC-3, 786-O, MDA-MB-231, and MCF-7 cell lines and microvesicle-associated fVIIa and TF antigen and activity levels were measured. Human coronary artery endothelial cells (HCAECs) were incubated with these purified microvesicles, or with combinations of fVIIa-recombinant TF, and cell proliferation/apoptosis was measured. Additionally, by expressing mCherry-PAR2 on HCAEC surface, PAR2 activation was quantified. Finally, the activation of PAR2 on HCAEC or the activities of TF and fVIIa in microvesicles were blocked prior to addition of microvesicles to cells. The purified microvesicles exhibited a range of fVIIa:TF ratios with HepG2 and 786-O cells having the highest (54:1) and lowest (10:1) ratios, respectively. The reversal from proapoptotic to proliferative was estimated to occur at a fVIIa:TF molar ratio of 15:1, but HCAEC could not be rescued at higher TF concentrations. The purified microvesicles induced HCAEC proliferation or apoptosis according to this ruling. Blocking PAR2 activation on HCAEC, or inhibiting fVIIa or TF-procoagulant function on microvesicles prevented the influence on HCAEC. Finally, incubation of HCAEC with recombinant TF resulted in increased surface exposure of fVII. The induction of cell proliferation or apoptosis by TF-positive microvesicles is dependent on the ratio of fVIIa:TF and involves the activation of PAR2. At lower TF concentrations, fVIIa can counteract the proapoptotic stimulus and induce proliferation.

## Introduction

Tissue factor (TF) initiates the coagulation mechanism through the formation of a complex with factor VIIa (fVIIa) which then activates factors X and IX. 1 2 TF is expressed on the surface of cells and may be released as cell-derived microvesicles following cellular activation. 3 4 5 6 7 8 9 TF is capable of initiating cellular signals in cells expressing this protein, and also on exposure of recipient cells to exogenous TF-containing microvesicles. TF signaling can alter the cellular gene expression profile 10 11 and has been demonstrated to include fVIIa activity and protease-activated receptor-2 (PAR2) activation. 12 13 14 15 16 17 18 Furthermore, interaction with β-integrins has also been implicated in inducing cell proliferation. 14 19 20 21 TF signaling is particularly associated with a high proliferative capacity in cancer cells. 22 23 24 However, while the proliferative potential has been associated with the interaction of TF with fVIIa, the data on the requirement for proteolytic function of fVIIa are not consistent. 12 13 We previously showed that the exposure of cells to low levels of recombinant TF alone promotes entry into the cell cycle. 25 However, the exposure of cells to high levels of TF additionally induces cell cycle arrest at the G1/S checkpoint and can lead to cell apoptosis. 25 26 27 In addition, monocyte-derived microparticles can induce cellular apoptosis in endothelial cells 28 and eosinophils lacking fVII become more susceptible to apoptosis. 29 Therefore, the magnitude of exposure to TF may itself determine the outcome in the recipient cells. Furthermore, the formation of TF–fVIIa complex is capable of triggering signals via PAR2 directly, or alternatively through the activation of factor Xa (fXa) and formation of a tertiary complex. 30 31 The activation of PAR2 has also been shown to be essential in the signaling processes that are initiated from the exposure of cells to TF. 32 33

It has been shown that as a consequence of inflammation, disease, or injury, large quantities of TF are released within microvesicles. 34 35 36 37 These microvesicles interact with endothelial cells and have also been shown to be cleared by endocytosis. 38 While the inability of cells to satisfactorily process TF is detrimental to endothelial cells, 27 acute exposure of cells to TF can also induce cellular apoptosis. 25 26 28 Therefore, prolonged exposure of the endothelial layer to TF-containing microvesicles may give rise to endothelial dysfunction and denudation as seen in chronic diseases. 6 39 40 Understanding the criteria by which TF-containing microvesicles function, and determining the characteristics that confer the proliferative and proapoptotic properties to the microvesicles, may lead to a new understanding of the relationship between various inflammatory diseases such as cancer and atherosclerosis with the onset and progression of vascular disease. In this study, it was hypothesized that the ratio of fVIIa:TF within cell-derived microvesicles is a determinant of the outcome of the exposure of endothelial cells to the microvesicles. Consequently, the ability of the fVIIa–TF complex, and cell-derived microvesicles containing a range of molar ratios of fVIIa:TF, to promote endothelial cell proliferation or alternatively apoptosis was evaluated.

## Material and Methods

### Cell Culture

Five cell lines were selected to include a range of TF expression and not on the basis of tissue of origin. BxPC-3 pancreatic cancer and 786-O renal carcinoma cell lines (ATCC, Teddington, United Kingdom) were cultured in RPMI-1640 medium, MCF-7 breast cancer cell line and HepG2 hepatocellular carcinoma cell lines (ATCC) were cultured in EMEM, and MDA-MB-231 (ATCC) breast cancer cell lines were cultured in DMEM. All media were supplemented with 10% (v/v) heat-inactivated fetal calf serum (FCS) to ensure the lack of any functional enzymes. Human coronary artery endothelial cells (HCAECs), devoid of endogenous TF, were cultured in MV media containing 5% (v/v) FCS and growth supplements (PromoCell, Heidelberg, Germany). In some experiments, the cells were adapted to serum-free medium prior to use.

### Preparation and Analysis of the TF-Containing Microvesicles

Cell lines were propagated in 25 cm ^2^ flasks, washed with phosphate-buffered saline (PBS) pH 7.4 and adapted to respective serum-free medium, for 1 hour prior to collection by ultracentrifugation according to described procedures for preparation and confirmation of the microvesicles. 38 41 The functional density of the released microvesicles was determined using the Zymuphen MP-assay kit (Hyphen BioMed/Quadratech, Epsom, United Kingdom) using the standards provided with the kit. The particle densities were also verified and the size distribution of microvesicles was also examined by nanoparticle tracking analysis using a Nanosight NTA 2.3 instrument. 38 41

### Qualitative Analysis of fVII/fVIIa and fX/fXa Antigens in the Microvesicles by Western Blot

The presence of fX and fVII antigen was detected by western blot analysis. The samples were separated by 12% (w/v) sodium dodecyl sulfate polyacrylamide gel electrophoresis (SDS-PAGE), transferred onto nitrocellulose membranes, blocked with TBST (10 mM Tris-HCl pH 7.4, 150 mM NaCl, 0.05% Tween-20). The membranes were then probed with a mouse monoclonal anti-fX antibody (156106), or a rabbit polyclonal anti-fVII antibody (both from R&D Systems, Abingdon, United Kingdom), both diluted 1:2000 (v/v) in TBST. The membranes were developed with alkaline phosphatase-conjugated goat antimouse or goat antirabbit antibodies (Santa Cruz, Heidelberg, Germany) respectively, diluted 1:4000 (v/v) and bands were visualized using the Western Blue stabilized alkaline phosphatase substrate (Promega, Southampton, United Kingdom).

### Quantitative Determination of TF and fVIIa Antigen Levels in the Microvesicles

The TF antigen associated with the microvesicles was measured using the Quantikine TF-ELISA kit (R&D Systems) and the factor VII/VIIa antigen levels were determined using the Assaymax FVII-ELISA kit (Assaypro/Universal Biologicals Ltd., Cambridge, United Kingdom) according to the manufacturers' instructions. To determine the cell surface fVIIa antigen in HCAEC without detachment, cells (2 × 10 ^4^ ) were treated as required and then fixed using 4% (v/v) formaldehyde. The cells were washed and one set was permeabilized while the other set was kept intact. Total and surface expression of fVII were measured in situ, by incubating all samples with a rabbit anti-fVIIa polyclonal antibody (10 µg/mL). The samples were then probed with a HRP-conjugated goat antirabbit antibody (dilute 1:1000 v/v) and developed using the TMB One-solution HRP substrate (Promega). The absorption values were measured at 450 nm from which the percentage ratio of surface:total fVII was calculated. In some experiments, the cells were subjected to repeated exposure to TF at 60 minute intervals and the cell surface fVII antigen was measured and calculated as a percentage of the original cell-surface fVII.

### Measurement of TF, fVIIa, and fXa Activity

Microvesicle TF-fVIIa activities were measured by modification of previously described procedures. 42 43 To measure TF activity, microvesicle samples were incubated with fVIIa (10 nM) in HEPES-buffered saline (HBS) pH 7.4, containing 1% (w/v) bovine serum albumin (BSA) and 5 mM CaCl _2_ , together with fX (100 nM) and a fXa chromogenic substrate (0.2 mM; Hyphen) diluted in the same buffer (200 µL). The samples were incubated for 60 minutes to develop the color. Aliquots (150 µl) were then transferred to a 96-well plate containing 2% (v/v) acetic acid (50 µL) and the absorptions measured immediately at 410 nm. The amount of fXa generated was determined using a standard curve prepared using fXa (0–20 nM; Enzyme Research Labs). To detect microvesicle-associated fVIIa activity, exogenous fVIIa was omitted from the above, and replaced with recombinant TF (1 U/mL). The absorption measurements were compared with a set of controls prepared using a range of fVIIa (0–10 nM) which were supplemented with recombinant TF (1 U/mL) and analyzed for fXa-generation potential as above. To examine the levels of fXa activity, the microvesicles were diluted in the buffer as above and incubated with the fXa substrate alone. The absorption measurements were compared with a set of controls prepared using a range of fXa as above.

### Treatment of HCAEC with fVII:TF and Microvesicles

HCAEC (2 × 10 ^4^ ) were incubated with microvesicles prepared from the conditioned serum-free media from the different cell lines, at microvesicle densities stated in the Results section. Other sets of cells were incubated with combinations of recombinant human TF (Innovin thromboplastin reagent; Dade Behring, Deerfield, United States) at a range of 0 to 10 U/mL (1 U/mL = 1.3 ng/mL), and/or purified fVIIa (Enzyme Research Labs, Swansea, United Kingdom) at a range of 0 to 10 nM. Cell numbers were determined at 24 hour, by staining with crystal violet as previously described and calculated from a standard curve. 38 As further confirmation of entry into the cell cycle, the expression of cyclin D1 mRNA was measured by reverse transcription polymerase chain reaction (RT-PCR) as previously described. 25 Total RNA was extracted using the Ribozol solution (VWR, Lutterworth, United Kingdom). The expression of cyclin D1 mRNA was measured by GoTaq 1-Step RT-qPCR System (Promega, Southampton, United Kingdom) using QuantiTect primers for cyclin D1 and β-actin (Qiagene, Manchester, United Kingdom) and relative amounts determined using as a reference. In addition, cellular apoptosis was quantified using the TiterTACS Colorimetric Apoptosis Detection Kit (AMS Biotechnology, Abingdon, United Kingdom) according to the manufacturer's instructions. 27 The data for apoptosis were expressed as the change in absorption measured at 450 nm. Furthermore, the expression of bax mRNA was quantified in the cell samples according to described procedures and using the reagents above, to further confirm the induction of cell apoptosis. 25 27 In some experiments, the microvesicles were preincubated with an inhibitory mouse antihuman TF antibody (HTF-1; 20 µg/mL; eBioscience/Thermo Scientific, Warrington, United Kingdom), a signal-blocking antihuman TF antibody (10H10; 20 µg/mL; BD Bioscience, Wokingham, United Kingdom), an inhibitory polyclonal rabbit antihuman fVIIa antibody (10 µg/mL; Abcam, Cambridge, United Kingdom), or respective control isotype IgG antibodies (20 µg/mL; New England Biolabs, Hitchin, United Kingdom). In other experiments, the activation of PAR2 on HCAEC was blocked by preincubation of cells with an inhibitory anti-PAR2 antibody (SAM11; Santa Cruz Biotechnology, Heidelberg, Germany) which was used at 20 µg/mL. Additionally, sets of HCAEC were supplemented with PAR2-activating peptide (PAR2-AP; SLIGKV) at a final concentration of 20 µM, simultaneously with the addition of the microvesicles (Severn Biotech Ltd, Kidderminster, United Kingdom). Finally, in some experiments the cells were preincubated with Rivaroxaban (pure compound supplied by Bayer, Leverkusen, Germany) and used at therapeutic concentrations (0.6 µg/mL), which was also optimized against fXa prior to this study. 44

### Preparation of mCherry-PAR2 Hybrid and Measurement of PAR2 Activation

To measure the activation of PAR2, a hybrid tandem protein was expressed to contain mCherry, followed by full-length PAR2. The hybrid protein was therefore designed so that the proteolytic activation of PAR2 released mCherry from the cell surface, into the medium. The cDNA for PAR2 was amplified by PCR from the hPAR2 VersaClone cDNA plasmid (R&D Systems) using the forward (5′-GCTCAAGCTTATCCAAGGAACCAATAGATCCTC) and reverse (5′-CGGTGGATCCTTAATAGGAGGTCTTAACAGTGG) primers and then digested with *Bam* HI and *Hind* III restriction enzymes (1 U/mL; New England Biolabs). The insert was ligated using the Instant Sticky-end ligase master mix (New England Biolabs), at a molar ratio of 3:1 into a mCherry2-C1 vector (Addgene) which was predigested with the same two restriction enzymes. 5α-Competent *Escherichia coli* bacteria were transformed with the plasmid DNA construct and colonies were selected from LB-agar plates containing carbenicillin (100 µg/mL). The cells were propagated and the positive colonies containing the mCherry-PAR2 construct were selected and confirmed by sequencing (Eurofins MWG, Wolverhampton, United Kingdom). Plasmid DNA was harvested from the bacteria by midi-prep (Promega Corp, Southampton, United Kingdom), using a procedure that eliminates any residual endotoxins. HCAECs were transfected with the mCherry-PAR2 constructs and surface expression was confirmed by fluorescence microscopy and also by comparing to cells labeled with FITC-conjugated anti-PAR2 antibody. The analysis was performed using a Zeiss Axio Vert.A1 inverted fluorescence microscope with a ×40 magnification (Carl Zeiss Ltd, Welwyn Garden City, United Kingdom). The activation of PAR2 was monitored by measuring the release of mCherry following the proteolytic digestion of PAR2. HCAECs expressing the hybrid protein were washed with PBS and adapted to serum-free medium. Since mCherry is multiply digested by the proteolytic action of fXa, the release of mCherry from the cell surface was only examined by incubation of cells with combinations of recombinant TF (0–4 U/mL) and purified fVIIa (0–4 nM) for up to 60 minutes. Finally, the ability of microvesicles derived from HepG2, BxPC-3, 786-O, MDA-MB-231, and MCF-7 cell lines, to release mCherry, was examined.

### Statistical Analysis

All data represent the calculated mean values from the number of experiments stated in each figure legend ± the calculated standard error of the mean. Statistical analysis was performed using the Statistical Package for the Social Sciences (SPSS Inc. Chicago, United States). Significance was determined using one-way ANOVA (analysis of variance) and Tukey's honest significance test or where appropriate, by paired *t* -test.

## Results

### The Ratio of fVIIa to TF Varies in Microvesicles Derived from Different Cell Lines

**Fig. 1:**
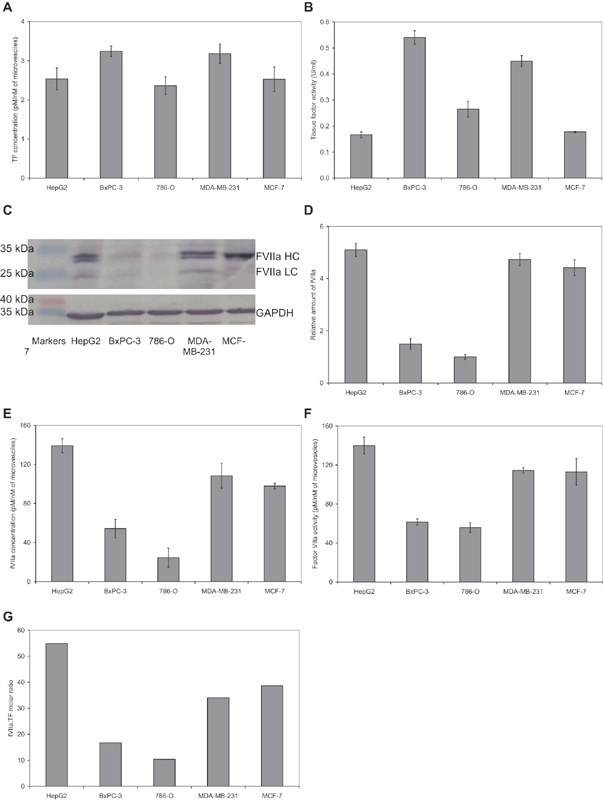
Analysis of the TF and fVIIa content of microvesicles. Five cell lines (HepG2, BxPC-3, 786-O, MDA-MB-231, and MCF-7) were propagated in 25 cm ^2^ flasks, washed with phosphate-buffered saline (PBS) pH 7.4 and adapted to respective serum-free medium, for 1 hour. Microvesicles were prepared from the conditioned media by ultracentrifugation and the functional density determined using the Zymuphen MP-assay kit. ( **A** ) The TF antigen concentration of the microvesicles was measured using the Quantikine TF-ELISA kit and ( **B** ) the associated TF activities were measured using the fXa-generation assay ( *n* = 5). ( **C** ) The presence of fVII/fVIIa antigen in the cells was detected by western blot analysis. The samples were separated by 12% (w/v) SDS-PAGE, transferred onto nitrocellulose membranes and then blocked with TBST (10 mM Tris-HCl pH 7.4, 150 mM NaCl, 0.05% Tween-20). The membranes were then probed with a rabbit polyclonal anti-fVII antibody diluted 1:2000 (v/v) in TBST and developed with an alkaline phosphatase-conjugated goat antirabbit antibody, diluted 1:4000 (v/v). The bands were visualized using the Western Blue stabilized alkaline phosphatase-substrate and ( **D** ) quantified by ImageJ program. (The micrographs are representative of three separate experiments). ( **E** ) Microvesicle-associated factor VII/VIIa antigen levels were quantified using the Assaymax FVII-ELISA kit ( *n* = 5). ( **F** ) Microvesicle-associated fVIIa activity was also measured by fXa-generation assay but in the absence of exogenous fVIIa and presence of TF (1 U/mL) ( *n* = 5). ( **G** ) The molar ratio of fVIIa:TF in microvesicles derived from the cells lines was calculated using the data generated above. SDS-PAGE, sodium dodecyl sulfate polyacrylamide gel electrophoresis; TF, tissue factor.

Throughout the study, microvesicles were purified from resting HepG2, BxPC-3, 786-O, MDA-MB-231, and MCF-7 cell lines in accordance to the diverse expression of TF, irrespective of tissue of origin. The microvesicles were purified by ultracentrifugation from serum-free conditioned media according to established procedures. 41 Prior to examining the influence of microvesicles on endothelial cell proliferation and apoptosis, the microvesicle density, TF antigen, fVII/fVIIa antigen, and fXa-generation potential of the microvesicles were analyzed. The particle densities were verified and the size distribution of microvesicles was also examined by NTA ( Supplementary Fig. S1 ). Comparison of similar quantities of microvesicles from the cells lines indicated that both BxPC-2 and MDA-MB-231 microvesicles contained the highest amounts of TF ( Fig. 1A ), which is in agreement with our previous study. 45 Examination of fXa-generation in the presence of supplemented fVIIa mostly reflected the measured TF antigen in the microvesicles ( Fig. 1B ). Examination of the fVIIa by western blot indicated the presence of active fVIIa in HepG2, MDA-MB-231, and MCF-7 cell lines, frequently exhibiting multiple bands ( Fig. 1C ) which have previously been attributed to the presence of glycosylation variants. 46 47 Moreover, 786-O and BxPC-3 cells contained relatively lower levels of fVIIa as measured by western blot ( Fig. 1D ). The amounts of fVIIa were also reflected in the relative levels of fVIIa mRNA expression in four of the cell lines ( Supplementary Fig. S2 ) but was four orders of magnitude higher in HepG2 cells (not shown). The amounts of microvesicle-associated fVII were then quantified using a fVII-ELISA kit ( Fig. 1E ) and were largely in line with those detected by western blot. In the absence of exogenous fVIIa, microvesicles from HepG2, MDA-MB-231, and MCF-7 cells possessed higher fXa-generation capacity indicating high fVIIa activity ( Fig. 1F ), which largely agreed with the level of fVII antigen in these microvesicles ( Fig. 1E ). In contrast, lower fVIIa activities were detectable in microvesicles from BxPC-3 and 786-O cell lines ( Fig. 1F ). Preincubation of all microvesicles with an inhibitory polyclonal anti-fVII antibody abolished the inherent fVIIa activity measured as above (not shown). The fVII and TF antigen quantities were then used to determine the ratio of fVIIa antigen to TF antigen and were established to be highest in HepG2 cells and lowest in 786-O cells ( Fig. 1G ). The presence of fVIIa on the surface of microvesicles requires calcium ions. It has been shown that microvesicles can contain a high concentration of calcium ions, 48 which is released during the formation of microvesicles by calcium-sensitive enzymes including calpains and gelsolins, and therefore permits the nonspecific binding of fVIIa to the microvesicle surface. Moreover, the concentration of fVIIa is only pathophysiologically relevant as fVIIa/TF complex and is therefore dictated by the concentration of TF and not fVIIa. However, none of the microvesicles examined contained fX/fXa antigen when examined by western blot, or possessed any detectable fXa activity following incubation with an fXa-specific chromogenic substrate (not shown).

**Fig. 2:**
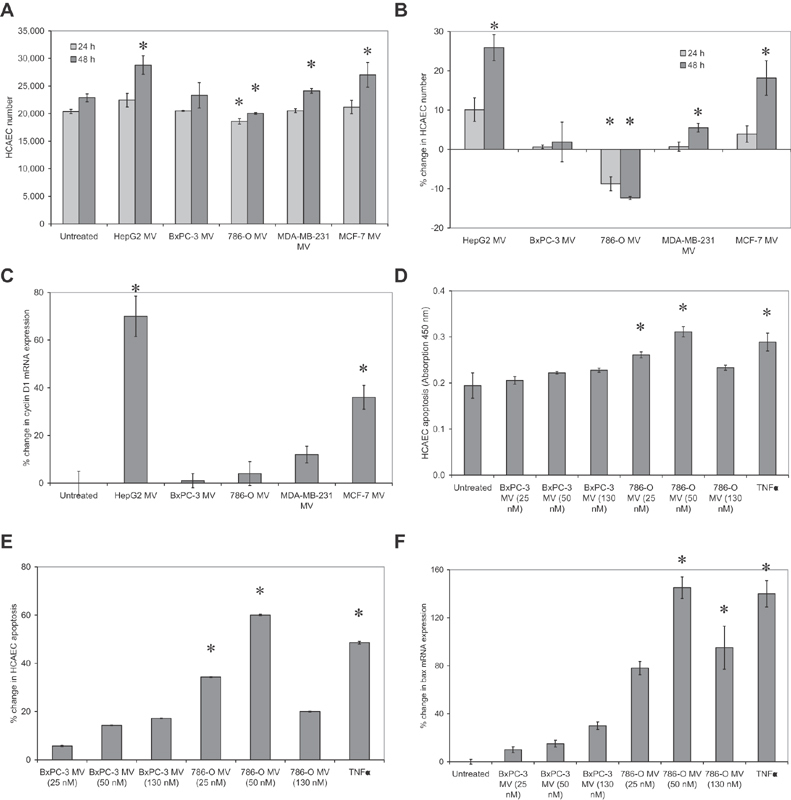
Examination of the influence of cell line-derived microvesicles on HCAEC cell numbers. Microvesicles were prepared from cell lines (HepG2, BxPC-3, 786-O, MDA-MB-231, and MCF-7), supplemented (50 nM) to HCAEC (2 × 10 ^4^ ) and incubated in for up to 48 hours. ( **A** ) Cell numbers were determined using the crystal violet staining assay and ( **B** ) the change in cell numbers was calculated as a percentage of the untreated cells ( *n* = 5; * = *p* < 0.05 vs. the respective untreated sample at each time point). ( **C** ) Total RNA was extracted from samples of the treated HCAEC and the relative expression of cyclin D1 mRNA was analyzed by RT-PCR against β-actin as housekeeping ( *n* = 3; * = *p* < 0.05 vs. the untreated sample). Cellular apoptosis was also measured (as absorption at 450 nm) in sets of HCAEC using the TiterTACS chromogenic TUNEL assay and ( **D** ) the change in level of apoptosis was calculated as a percentage of the untreated cells ( *n* = 5; * = *p* < 0.05 vs. the untreated sample). ( **E** ) Total RNA was extracted from samples of the treated HCAEC and the relative expression of bax mRNA was analyzed by RT-PCR against β-actin as housekeeping ( *n* = 3; * = *p* < 0.05 vs. the untreated sample). HCAEC, human coronary artery endothelial cell; RT-PCR, reverse transcription polymerase chain reaction.

### Cell Derived Microvesicles Differentially Influence Endothelial Cells

The microvesicles derived from the cell lines were incubated with HCAEC and the influence on cell proliferation or apoptosis was measured. Examination of HCAEC cell numbers indicated that the greatest increases in cell numbers on incubation with microvesicles are derived from HepG2 and MCF-7 cells ( Fig. 2A and 2B ). In addition, marginal increases were observed with microvesicles from MDA-MB-231 cells, but not BxPC-3 cells. These increases in cell numbers were also reflected in the expression of cyclin D1 as a marker for cell-cycle entry ( Fig. 2C ). In contrast, supplementation of HCAEC with microvesicles derived from 786-O resulted in reduction in cell numbers compared with the untreated sample. The next sets of experiments examining the induction of apoptosis only were then focused onto microvesicles derived from 786-O cells. These were compared with the outcome from microvesicles derived from BxPC-3 cells which had little influence. HCAECs were incubated with three microvesicle densities (25–130 nM) from each cell line. The rate of HCAEC apoptosis was then measured using a chromogenic TUNEL assay. Incubation of HCAEC with 50 nM of 786-O cell-derived microvesicles resulted in maximal level of cell apoptosis ( Fig. 2D and 2E ) but was not significant with BxPC-3-derived microvesicles. To further confirm the increases in cellular apoptosis, the expression of bax mRNA was measured and shown to agree with the data using the TUNEL assay ( Fig. 2F ).

### The Ratio of fVIIa:TF within Microvesicles Determines the Outcome on Endothelial Cells

**Fig. 3:**
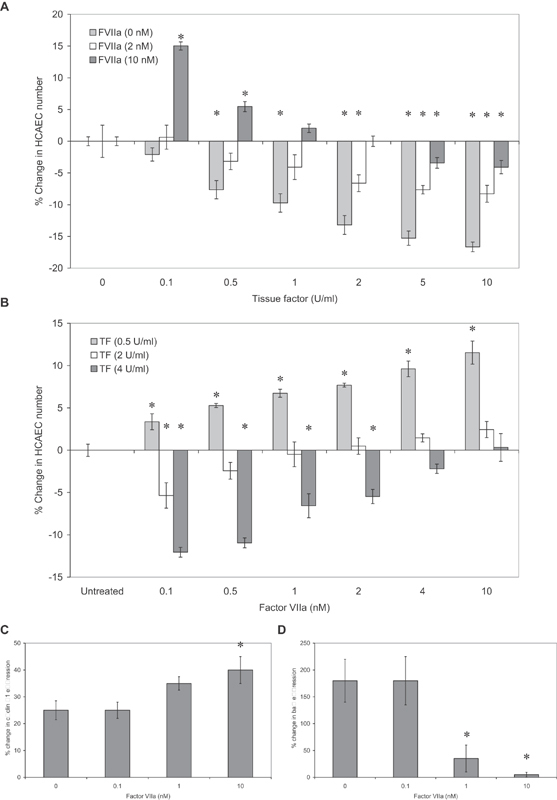
Examination of the effect of different ratios of fVIIa:TF on HCAEC cell numbers. ( **A** ) HCAECs (2 × 10 ^4^ ) were incubated for 24 hours with a range of recombinant human TF (Innovin thromboplastin reagent) at a range of 0–10 U/mL (1 U/mL = 1.3 ng/mL), in the presence or absence of purified fVIIa (0–10 nM). Cell numbers were determined by crystal violet staining assay and percentage change in cell numbers calculated ( *n* = 5; * = *p* < 0.05 vs. the untreated sample). ( **B** ) HCAECs (2 × 10 ^4^ ) were also incubated for 24 hours with a range of concentrations of purified fVIIa (0.1–10 nM) in the presence of recombinant human TF (0–4 U/mL). Cell numbers were determined by crystal violet staining assay and percentage change in cell numbers calculated ( *n* = 5; * = *p* < 0.05 vs. the untreated sample). Total RNA was extracted from samples of the treated HCAEC and the relative expression of ( **C** ) cyclin D1 mRNA and ( **D** ) bax mRNA was analyzed by RT-PCR against β-actin as housekeeping ( *n* = 3; * = *p* < 0.05 vs. the untreated sample). HCAEC, human coronary artery endothelial cells; RT-PCR, reverse transcription polymerase chain reaction; TF, tissue factor.

To further examine the relevance of microvesicle-associated fVIIa and TF, sets of HCAECs were incubated with combinations of recombinant TF (0–10 U/mL; Innovin), in conjunction with three concentrations of fVIIa (0–10 nM). In the absence of fVIIa, incubation of cells with recombinant TF resulted in lowering of cell numbers ( Fig. 3A ). Supplementation with fVIIa (2 nM) partially prevented the decline in cell numbers but became ineffective at TF concentrations higher than 0.5 U/mL. Inclusion of 10 nM fVIIa restored the cell numbers up to 2 U/mL of TF and was proliferative when combined together with lower TF concentrations. It is also noteworthy that in the absence of TF the addition of fVIIa had no detectable effect. These data were also confirmed by titrating a range of concentrations of fVIIa, together with three separate concentrations of TF. On supplementing the cells with 0.5 U/mL of TF, increased HCAEC numbers were observed with all the concentrations of fVIIa tested ( Fig. 3B ). However, in the presence of 2 U/mL of TF, fVIIa concentrations of 1 nM or higher were required to preserve or increase the cell numbers. Furthermore, on addition of 4 U/mL of TF, only inclusion of 10 nM fVIIa was capable of preventing the reduction in cell numbers. Analysis of cyclin D1 and bax expression in HCAEC treated with recombinant TF (2 U/mL) and purified fVIIa (0–10 nM) indicated entry into the cell cycle regardless of fVIIa concentration, in agreement with our previous findings, 25 but was highest at 10 nM ( Fig. 3C ), while bax expression was only detectable at lower fVIIa concentrations ( Fig. 3D ).

### Microvesicle-Induced Cell Proliferation and Apoptosis is Mediated through PAR2

**Fig. 4:**
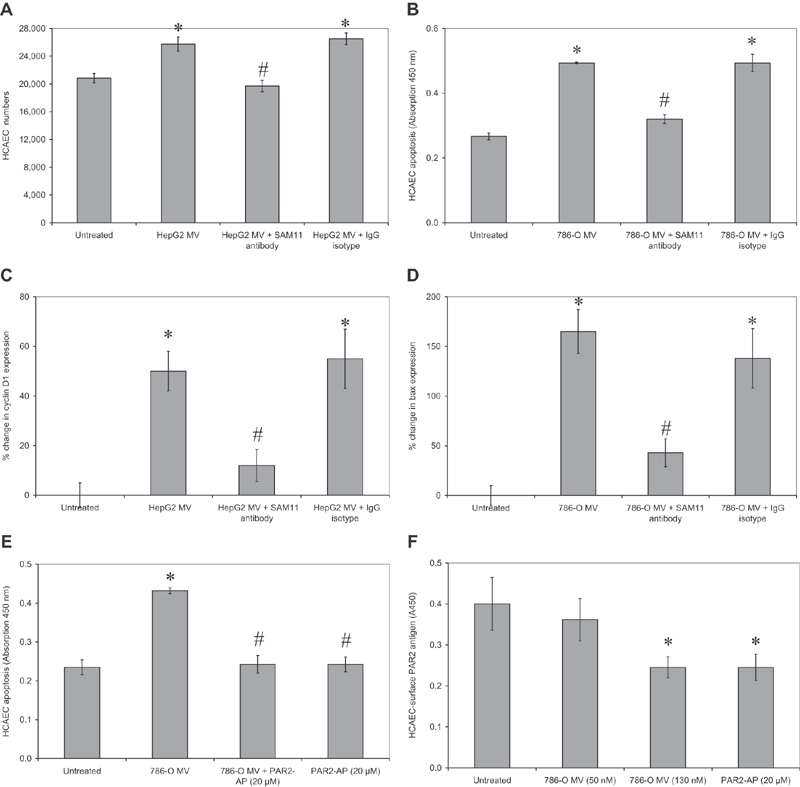
Examination of the involvement of PAR2 in microvesicle-induced HCAEC apoptosis. Sets of HCAEC (2 × 10 ^4^ ) were preincubated with a blocking anti-PAR2 antibody (SAM11; 20 µ/mL) or a control isotype. Sets of cells were then incubated with microvesicles derived from ( **A** ) HepG2 cell line (50 nM) for 24 hours and cell numbers were determined by crystal violet staining assay ( *n* = 5; * = *p* < 0.05 vs. the untreated sample, # = *p* < 0.05 vs. the sample devoid of antibody). ( **B** ) A similar set of cells was incubated for 24 hours with microvesicles derived from 786-O cell line (50 nM) and the rate of apoptosis determined using the TiterTACS chromogenic TUNEL assay ( *n* = 5; * = *p* < 0.05 vs. the untreated sample, # = *p* < 0.05 vs. the sample devoid of antibody). Total RNA was extracted from samples of the treated HCAEC and ( **C** ) the relative expression of cyclin D1 mRNA, and ( **D** ) bax mRNA was analyzed by RT-PCR against β-actin as housekeeping ( *n* = 3; * = *p* < 0.05 vs. the untreated sample, # = *p* < 0.05 vs. the sample devoid of antibody). ( **E** ) HCAECs (2 × 10 ^4^ ) were incubated for 24 hours with microvesicles derived from 786-O cell line (50 nM) in the presence or absence of PAR2-activating peptide (20 µM) and the rate of apoptosis determined ( *n* = 5; * = *p* < 0.05 vs. the untreated sample, # = *p* < 0.05 vs. microvesicles only). ( **F** ) HCAECs (2 × 10 ^4^ ) were incubated for 30 minutes with microvesicles derived from 786-O cell line (0–130 nM), or PAR2-activting peptide (20 µM). The cells were then incubated with a mouse antihuman PAR2 antibody (SAM11; 20 µg/mL) and probed with a HRP-conjugated goat antimouse antibody diluted 1:1000 (v/v). The relative amount of cell-surface PAR2 was then measured using the TMB substrate ( *n* = 5; * = *p* < 0.05 vs. the untreated sample). HCAEC, human coronary artery endothelial cells; PAR2, protease-activated receptor-2; RT-PCR, reverse transcription polymerase chain reaction.

To explore the mechanisms involved in the induction of cell proliferation and apoptosis by microvesicles, HCAECs were preincubated with an antibody to prevent PAR2 activation (SAM11; 20 µg/mL), prior to addition of microvesicles derived from HepG2 or 786-O cell lines. Inhibition of PAR2 activation prevented the increase in HCAEC numbers in response to microvesicles derived from HepG2 cell line, after 24 hours ( Fig. 4A ). However, preventing the activation of PAR2 using the antibody also suppressed the proapoptotic influence of 786-O cell-derived microvesicles ( Fig. 4B ). Moreover, the analysis of cyclin D1 expression in HCAEC stimulated with microvesicles derived from HepG2 cells ( Fig. 4C ) or bax mRNA in cells stimulated with microvesicles derived from 786-O cells ( Fig. 4D ) confirmed the requirement for PAR2 activation in both the proliferative and proapoptotic outcomes. Preincubation of 786-O microvesicles, or alternatively the HCAEC with effective concentrations of Rivaroxaban (0.6 µg/mL) to inhibit any present fXa, did not prevent endothelial apoptosis (not shown). Moreover, simultaneous activation of PAR2 using the activating peptide (SLIGKV; 20 µM) in conjunction with 786-O cell-derived microvesicles (50 nM) was capable of rendering these microvesicles ineffective ( Fig. 4E ). To decipher these observations, the presence of PAR2 antigen on the surface of cell surface, following the addition of PAR2-AP or a range of concentrations of microvesicles from 786-O cells, was examined. The HCAECs were probed with a PAR2 antibody in situ, to avoid nonspecific activation of PAR2 by trypsinization. HCAECs were incubated with microvesicles from 786-O cells at the optimal (50 nM) and hyper-optimal (130 nM) densities to induce apoptosis ( Fig. 2E ). Incubation of HCAEC with PAR2-AP (20 µM) or with 130 nM of 786-O cell-derived microvesicles resulted in significant reductions in cell surface PAR2 within 30 minutes ( Fig. 4F ). In contrast, cell-surface PAR2 levels remained unaltered by the addition of 50 nM of these microvesicles.

### Microvesicles Differentially Activate PAR2 on the Surface of Endothelial Cells

**Fig. 5:**
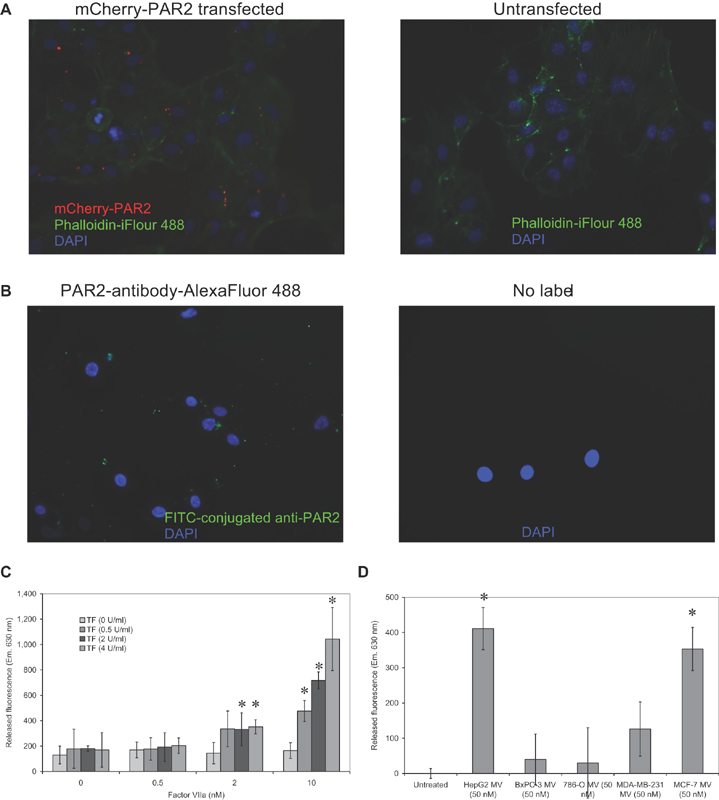
Examination of the activation of PAR2 in response to fVIIa:TF complex and microvesicles. Sets of HCAEC (5 × 10 ^3^ ) were transfected to express mCherry-PAR2 hybrid protein. ( **A** ) Sets of transfected and untransfected cells were then fixed and stained phalloidin-iFlour 488 (diluted 1:1000 v/v) and DAPI (2 µg/mL) and examined by fluorescence microscopy for mCherry-PAR2 (Red), phalloidin-iFlour 488 (Green), and DAPI (Blue). ( **B** ) As a comparison, untransfected cells which were labeled with FITC-conjugated anti-PAR2 (SAM11; 20 µg/mL) or unlabeled cells. The cells were examined by fluorescence microscopy for PAR2 (Green) and DAPI (Blue). ( **C** ) HCAECs expressing the mCherry-PAR2 protein were incubated for 1 hour with combinations of TF (0–4 U/mL) together with fVIIa (0–10 nM) and the release of mCherry in the media was measured by determining the fluorescence at Em. 630 nm (Ext. 580 nm) ( *n* = 5; * = *p* < 0.05 vs. the untreated sample). ( **D** ) Sets of transfected cells were also incubated with microvesicles derived from the five cell lines (HepG2, BxPC-3, 786-O, MDA-MB-231, and MCF-7) at 50 nM and the release of mCherry measured by determining the fluorescence at Em. 630 nm (Ext. 580 nm) ( *n* = 5; * = *p* < 0.05 vs. the untreated sample). HCAEC, human coronary artery endothelial cells; PAR2, protease-activated receptor-2; TF, tissue factor.

To examine the level of PAR2 activation in response to various microvesicles, a hybrid construct was prepared containing the cDNA for mCherry-PAR2 and was expressed in HCAEC. The expression of the mCherry-PAR2 in HCAEC was confirmed by fluorescence microscopy ( Fig. 5A ). This was then compared with the pattern of native PAR2 expression, probed using Alexa Fluor488-conjugated anti-PAR2 (SAM11) antibody ( Fig. 5B ). To determine the effectiveness of the TF–fVIIa complex in activating PAR2, the transfected cells were incubated with combinations of TF (0–4 U/mL) together with fVIIa (0–10 nM) and the release of mCherry in the media was measured at Em. 630 nm (Ext. 580 nm). In the absence of TF, the addition of fVIIa (0–10 nM) did not result in the release of fluorescence into the media ( Fig. 5C ). Moreover, addition of TF alone, or together with low level of fVIIa (0.5 nM), was not sufficient to induce PAR2 activation. Incubation of cells with TF together with fVIIa (2 nM) resulted in marginal increases in fluorescence, which was significant at higher TF concentrations. In contrast, inclusion of fVIIa (10 nM) resulted in a TF concentration-dependent activation of PAR2 from the transfected HCAEC. Examination of the effectiveness of the microvesicles purified from the five cell lines in activating mCherry-PAR2 expressed on the surface endothelial cell confirmed a high activity associated with HepG2 and MCF-7-derived microvesicles, with lower levels with MDA-MB-231 microvesicles but no significant PAR2 activation with microvesicles from BxPC-3 or 786-O cells ( Fig. 5D ).

### Exogenous fVIIa Prevents TF-Microvesicle-Induced Apoptosis

**Fig. 6:**
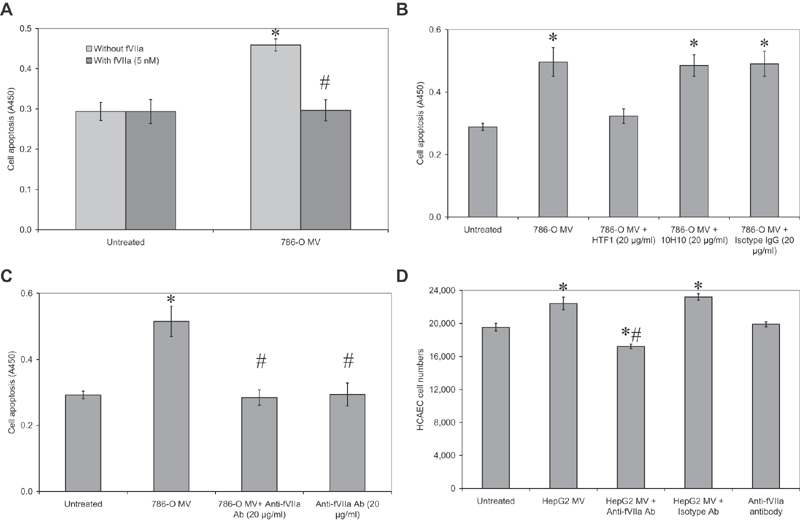
Active fVIIa both attenuates and is required for the induction of HCAEC apoptosis by microvesicle-associated TF. ( **A** ) Sets of HCAEC (2 × 10 ^4^ ) were incubated with microvesicles derived from 786-O cells alone, or supplemented with fVIIa (5 nM) and cellular apoptosis was measured after 24 hours ( *n* = 5; * = *p* < 0.05 vs. the untreated sample, # = *p* < 0.05 vs. the respective sample without fVIIa). ( **B** ) Microvesicles derived from 786-O cells were preincubated with HTF-1 anti-TF antibody (20 µg/mL) to block fVIIa binding, 10H10 anti-TF antibody (20 µg/mL) to block TF signaling, or an isotype mouse antibody (20 µg/mL), and cellular apoptosis was measured after 24 hours ( *n* = 5; * = *p* < 0.05 vs. the untreated sample). ( **C** ) Microvesicles derived from 786-O cells were preincubated with an inhibitory anti-fVIIa antibody (20 µg/mL) prior to addition to HCAEC (2 × 10 ^4^ ) and cellular apoptosis was measured after 24 hours ( *n* = 5; * = *p* < 0.05 vs. the untreated sample, # = *p* < 0.05 vs. sample with microvesicle alone). ( **D** ) Microvesicles derived from HepG2 cells were preincubated with an inhibitory anti-fVIIa antibody (20 µg/mL) prior to addition to HCAEC (2 × 10 ^4^ ). HCAEC numbers were determined after 24 hours using the crystal violet staining assay and the cell numbers calculated ( *n* = 5; * = *p* < 0.05 vs. the untreated sample, # = *p* < 0.05 vs. sample with microvesicle alone). HCAEC, human coronary artery endothelial cells; TF, tissue factor.

To test the hypothesis that increased levels of fVIIa can rescue the cells from microvesicle-induced apoptosis, and may induce proliferation, HCAECs were incubated with 786-O cell-derived microvesicles in the presence or absence of exogenous fVIIa (2 nM). Inclusion of additional fVIIa abolished the proapoptotic influence of 786-O cell-derived microvesicles ( Fig. 6A ). However, as shown before ( Fig. 3A ), the supplementation with fVIIa in the absence of any TF did not influence the HCAEC numbers which are otherwise devoid of endogenous TF expression. Preincubation of 786-O microvesicles with an inhibitory anti-TF antibody (HTF-1; 20 µg/mL), which blocks the procoagulant activity of TF, prevented cellular apoptosis ( Fig. 6B ). However, preincubation of these microvesicles with the 10H10 anti-TF antibody, which inhibits TF signaling, or the isotype control antibody did not interfere with the promotion of apoptosis in HCAEC. In addition, neutralization of fVIIa in microvesicles derived from 786-O and HepG2 cells, using an inhibitory anti-fVIIa antibody, prevented the promotion of cell apoptosis in response to 786-O-derived microvesicles ( Fig. 6C ) and also the increased cell numbers by HepG2-derived microvesicles ( Fig. 6D ), respectively. 10H10 antibody blocks TF signaling by blocking TF interaction with integrins 49 50 and the secondary/exosite PAR2-binding site within TF. 51 However, unlike HTF1, it does not prevent the interaction with fVIIa, 51 nor does it affect the proteolytic activity of the TF/fVIIa complex. 52 Therefore, in agreement with the data using the inhibitory anti-fVIIa antibody ( Fig. 6C ), the initiation of the described mechanism is dependent on the proteolytic activity of fVIIa as well as requires TF.

### Endothelial Cells Release fVII in Response to TF

**Fig. 7:**
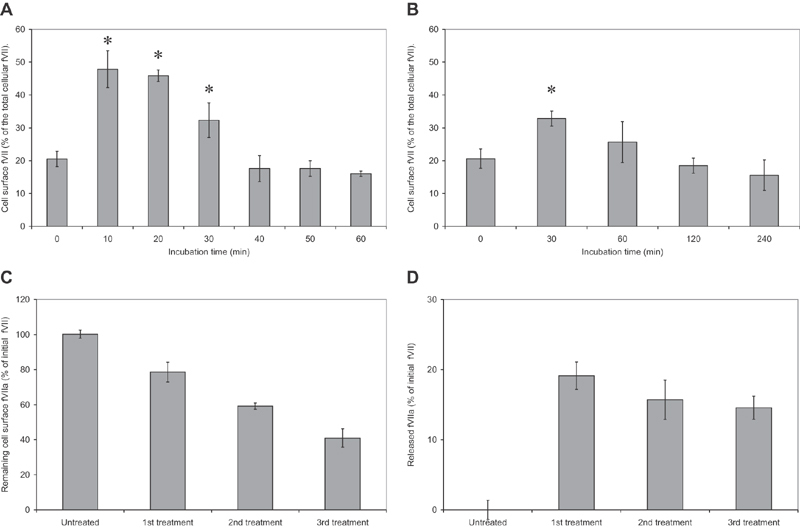
HCAECs express fVIIa on the cell surface in response to TF and PAR2 activation. Two separate sets of HCAEC (2 × 10 ^4^ ) were incubated with ( **A** ) TF (2 U/mL) or ( **B** ) PAR2-activating peptide (20 µM) for the durations shown and the cells were then fixed using 4% (v/v) formaldehyde. The cells were washed and one set was permeabilized while the other kept intact. Total and surface expression of fVII were measured in situ, by incubating all samples with a mouse anti-fVIIa antibody (20 µg/mL). The samples were then probed with a HRP-conjugated goat antimouse antibody (dilute 1:1000 v/v) and developed using the TMB substrate. The percentage ratio of surface:total fVII was then calculated ( *n* = 5; * = *p* < 0.05 vs. the observed ratio at time zero). HCAECs (2 × 10 ^4^ ) were also subjected to repeated treatment with recombinant TF (2 U/mL) at 60 minute intervals. After each treatment, the relative amount of ( **C** ) cell-surface fVII antigen and ( **D** ) the released microvesicle-associated fVII antigen were measured and the percentages were calculated against the amount of cell-surface fVII in the untreated cells. HCAEC, human coronary artery endothelial cells; PAR2, protease-activated receptor-2; TF, tissue factor.

Examination of HCAEC by RT-PCR indicated the presence of detectable amounts fVII mRNA in HCAEC (not shown). Measurement of the fVII protein lying intact and lysed HCAEC indicated that approximately 20% of the fVII was exposed at the surface of the resting cells ( Fig. 7A ). The presence of fVII associated with caveolae and Weibel–Palade bodies in differentiating endothelial cells has been demonstrated 53 and our unpublished data have shown some association of fVII with caveolae in resting endothelial cells (Madkhali et al, unpublished data). However, stimulation of HCAEC with recombinant TF (2 U/mL) resulted in a significant increase in cell surface fVII antigen (48% of the cellular fVII), as measured by the in situ labeling. Furthermore, activation of HCAEC with PAR2-AP (20 µM) resulted in a comparable but longer-term increase (33% of the cellular fVII at 30 minutes) in the concentration of cell-surface fVII ( Fig. 7B ). In some experiments, HCAECs were subjected to repeated treatment of cells with recombinant TF at 60 minute intervals. In these experiments, both the remaining cell-surface fVII and the microvesicle-associated fVII antigens were measured as a percentage of that present on the surface of the resting cells. Treatment of HCAEC with recombinant TF progressively decreased the amount of fVII present on the cell surface ( Fig. 7C ). Furthermore, the magnitudes of these reductions were accounted for by the amount of fVII that was associated with the released microvesicles ( Fig. 7D ).

## Discussion

The exposure of TF at the site of injury acts to initiate the coagulation mechanism and contains bleeding. However, as a factor which appears early at the sight of injury, TF is ideally placed to have a dual function in instructing the cells to divide or become apoptotic. The distinction between the severely injured cells and those which may be revived is imperative in the precise vascular homeostasis. These functional properties of TF also appear to be replicated in the microvesicle-associated TFs that are released into the bloodstream from various sources. In addition to TF, these microvesicles may also contain a complement of negatively charged phospholipids 3 4 5 6 7 8 and functional fVIIa ( Fig. 1 ). It is known that microvesicles from different sources exert dissimilar influence on endothelial cells, which provides crucial clues for the understanding of the destructive influence of microvesicles in diseases. 33 34 35 36 Therefore in this study, by measuring the ratios of fVII/fVIIa and TF, we examined a possible mechanism by which TF-containing microvesicles may confer different outcomes in cultured primary endothelial cells. In agreement with this hypothesis, incubation of HCAEC with combinations of purified fVIIa and recombinant TF resulted in different cellular outcomes depending on the fVIIa:TF ratio. The transition from the proapoptotic to proliferative property appears to occur at an estimated fVIIa:TF molar ratio of 15:1. This was also in agreement with the ratios observed in the microvesicles purified from the cell lines. Particularly, the fVIIa:TF ratio in the 786-O renal carcinoma cell line was 10:1 and these microvesicles induced cellular apoptosis in HCAEC ( Fig. 1G ). Higher molar ratios of 54:1and 38:1 observed in the HepG2 hepatocellular line and MCF-7 breast cancer line ( Fig. 1 ) were concurrent with increased HCAEC proliferation. Interestingly, the change in cell numbers was significantly proportional to the observed fVIIa:TF ratio (Pearson correlation = 0.956; *p* = 0.011). However, it also appears that as well as the fVIIa:TF ratio, the concentration of TF with which the cell comes into contact with is an additional critical factor in determining the outcome. Therefore, despite the similar fVIIa:TF molar ratios, microvesicles derived from MDA-MB-231 (34:1) were significantly less proliferative than those derived from MCF-7 cell lines (38:1). This may therefore be explained by the much higher TF content of microvesicles from MDA-MB-231 cells.

Although HCAEC lacked any detectable cell-surface TF, a significant proportion of cellular fVII was detected on the surface of these endothelial cells ( Fig. 7 ). Furthermore, activation of HCAEC with recombinant TF or PAR2-AP resulted in the exposure of a higher proportion of the cellular fVII. Therefore, it is possible that endothelial cells respond to the stimulatory signals arising from injury/trauma by altering the fVIIa:TF ratio, to counter the proapoptotic influence that arises from the presence of excessive levels of TF. However, because in our studies only the amounts of exogenous fVIIa and TF were used in calculating the fVIIa:TF molar ratios, the real ratios for the transition from the proapoptotic to proliferative form are likely to be higher than those reported here (15:1). In addition, repeated exposure of HCAEC resulted in the depletion of cellular fVII reserves ( Figs. 7C and 7D ). Such a compromise in endothelial cell function implies that the response by these cells may become insufficient in ensuring the survival of the cell. Therefore, we hypothesize that repeated exposure to TF-positive microvesicles, for example during chronic disease, exhausts the ability of endothelial cells to counter the proapoptotic property of TF.

To further elucidate the underlying proliferative mechanisms, PAR2, TF, and fVIIa were in turn inhibited using antibodies. Inhibition of PAR2 prevented the proapoptotic function of these microvesicles, indicating the requirement for PAR2 activation. Moreover, overstimulation of the cells with high concentrations of microvesicles or the addition of PAR2-AP was less effective due to the endocytosis of PAR2 and desensitization of HCAEC. Both the proliferative and proapoptotic influences of microvesicles were attributed to the proteolytic activity of the fVIIa-TF complex and were blocked by respective inhibitory antibodies. Finally, preincubation of microvesicles or HCAEC with Rivaroxaban to inhibit fXa did not prevent apoptosis in HCAEC. Microvesicles derived from different cell lines may contain pro- and antiapoptotic/proliferative material which can then contribute to endothelial cell proliferation or apoptosis. However, by selectively inhibiting TF or fVIIa ( Fig. 6 ), or alternatively blocking PAR2 ( Fig. 4 ), using specific antibodies we have demonstrated that these outcomes were initiated from the fVIIa/TF complex. In addition, the use of recombinant TF and purified fVIIa further confirmed the role of TF and fVIIa observed with cell-derived microvesicles ( Fig. 3 ). In contrast, activation of PAR2 in the absence of TF has been shown not to induce a similar complement of the signaling pathway and does not lead to apoptosis. 27

This study has for the first time shown that the ratio of fVIIa:TF determines the outcome in endothelial cells resulting in either proliferation or apoptosis. This is particularly relevant in the case of TF-containing microvesicles which are released during inflammatory conditions. The induction of cell proliferation and apoptosis by microvesicles appears to be mediated through the activation of PAR2, but the cellular outcome is entirely dependent on the amount of TF protein and the molar ratio of fVIIa-TF protease activity. However, the downstream events are only partially elucidated. In conclusion, a novel measurable parameter within procoagulant microvesicles that can determine the function of microvesicles during the activation of the vasculature has been identified.
