# Supplementary material for: The Ratio of Factor VIIa:Tissue Factor Content within Microvesicles Determines the Differential Influence on Endothelial Cells
Source: TH Open. 2019 May 15;3(2):e132–45. doi: 10.1055/s-0039-1688934 (PMC6598090; doi:10.1055/s-0039-1688934)
Supplement: Supplementary file 1 — Supplementary Material [file 10-1055-s-0039-1688934-s190014.pdf]

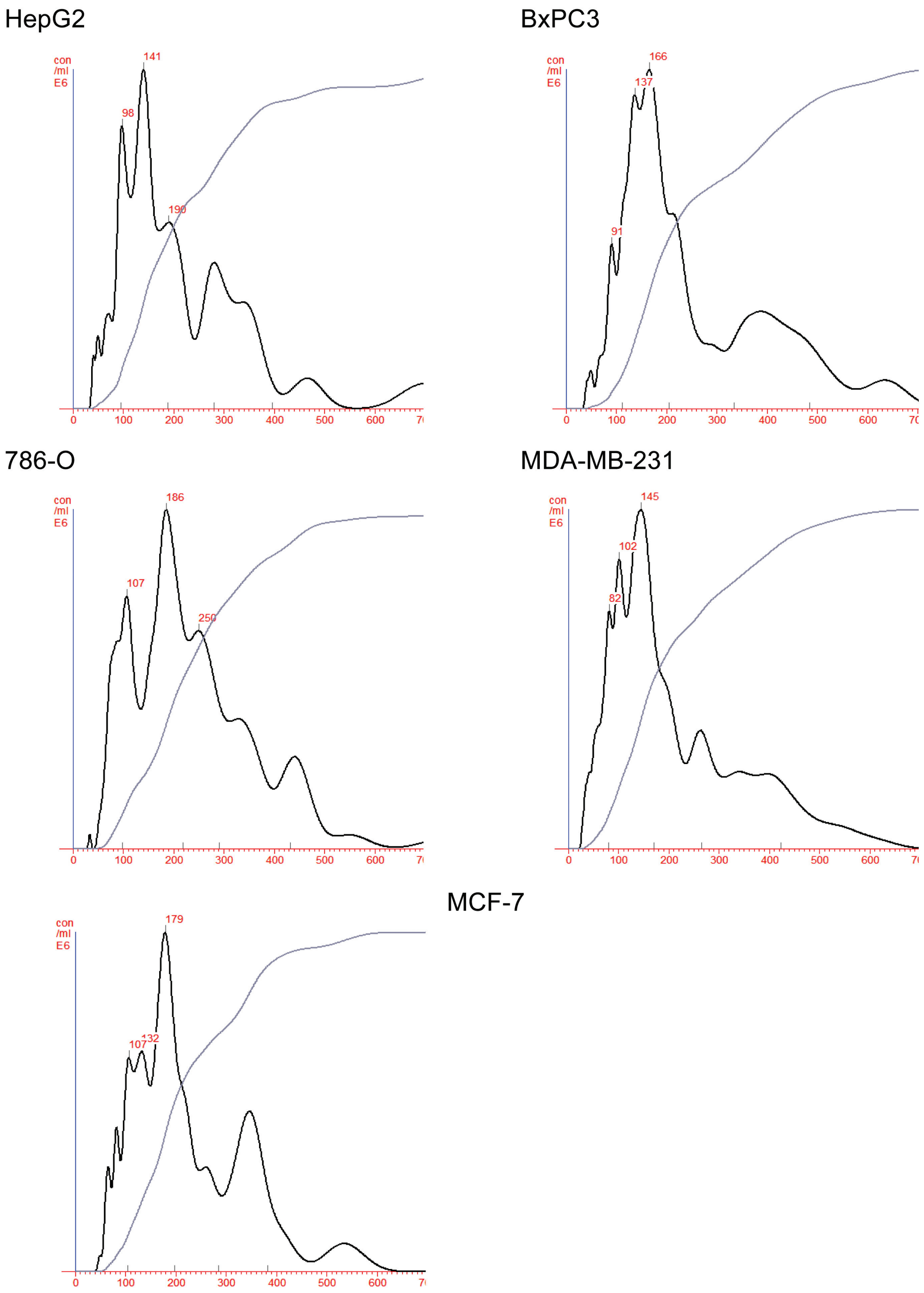

Supplementary Fig. S1 Microparticles were isolated from the conditioned media of HepG2, BxPC-3, 786-O, MDA-MB-231, or MCF-7 cells by ultracentrifugation at 100,000 *g* for 1 hour at 20°C, and resuspended in 0.1 µm-filtered PBS. The size distributions of the microvesicles were determined using a Nanosight NTA 2.3 instrument.

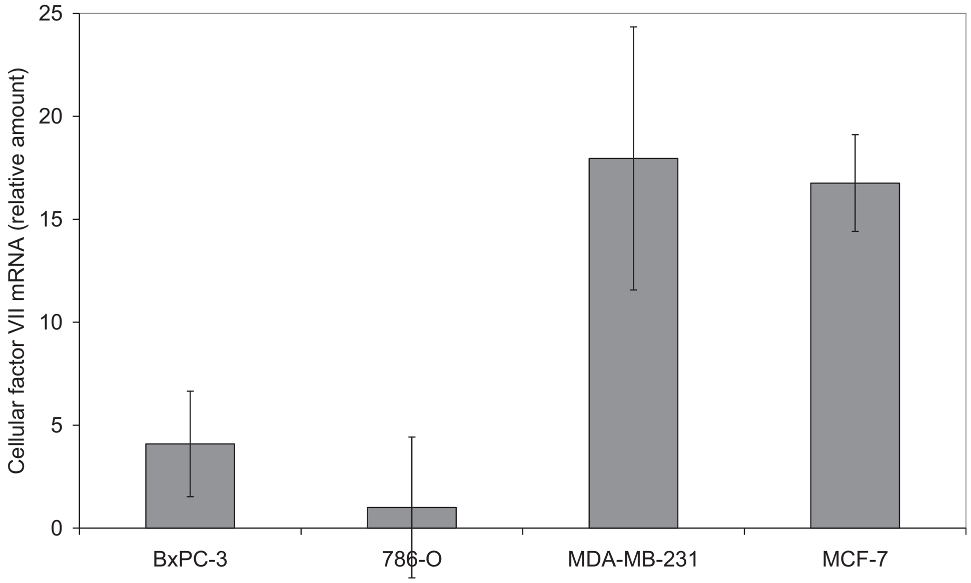

Supplementary Fig. S2 Five cell lines (BxPC-3, 786-O, HepG2, MDA-MB-231, and MCF-7) were propagated in 25 cm^2 flasks, washed with phosphate-buffered saline (PBS) pH 7.4. Cells (2×10^5) were centrifuged and the RNA was extracted using the Ribozol solution (VWR, Lutterworth, United Kingdom). The expression of fVIIa mRNA was measured by GoTaq 1-Step RT-qPCR System (Promega, Southampton, United Kingdom) using QuantiTect primers for human fVII and β-actin (Qiagene, Manchester, United Kingdom) and relative amounts determined using as a reference ($n = 3$).
